# In Vitro Evaluation of Accuracy of CBCT-Derived Volumes in Maxillary Defects: Effects of kVp, Device, and Software

**DOI:** 10.3390/diagnostics15101247

**Published:** 2025-05-14

**Authors:** Sema Murat, Kıvanç Kamburoğlu, Diego Vazquez, Leonardo Jorge Nart, Victoria Azcona, Lorena Elizabeth Benitez, Mohammed Awawdeh, Wael Aboelmaaty

**Affiliations:** 1 Department of Prosthodontics, Faculty of Dentistry, Ankara University, Ankara 06560, Turkey; semamurat47@yahoo.com.tr; 2 Department of Dentomaxillofacial Radiology, Faculty of Dentistry, Ankara University, Ankara 06560, Turkey; dtkivo@yahoo.com; 3 Department of Diagnostic Imaging, Faculty of Dentistry, University of Buenos Aires, Buenos Aires C1122AAH, Argentina; diego.vazquez@odontologia.uba.ar (D.V.); leonardo.nart@odontologia.uba.ar (L.J.N.); vicky.azcona@gmail.com (V.A.); lorena.benitez@odontologia.uba.ar (L.E.B.); 4 Department of Surgery and Pediatric Dentistry, Faculty of Stomatology, Khoja Akhmet Yassawi International Kazakh Turkish University, Turkestan 161200, Kazakhstan; 5 Preventive Dental Science Department, College of Dentistry, King Saud bin Abdulaziz University for Health Sciences (KSAU-HS), Riyadh 11481, Saudi Arabia; awawdehm@ksau-hs.edu.sa; 6 King Abdullah International Medical Research Center, Ministry of National Guard—Health Affairs, Riyadh 11481, Saudi Arabia; 7 Maxillofacial Surgery and Diagnostic Sciences Department, College of Dentistry, King Saud bin Abdulaziz University for Health Sciences (KSAU-HS), Riyadh 11481, Saudi Arabia; 8 Oral Radiology and Diagnostic Sciences Department, Faculty of Dentistry, Mansoura University, Mansoura City 35516, Egypt

**Keywords:** cone-beam computed tomography, X-ray microtomography, image processing, computer-assisted, volume measurement

## Abstract

**Objectives:** This study aimed to evaluate the accuracy of CBCT-based volumetric measurements of maxillary defects and to investigate the effects of different CBCT devices, kVp settings, and segmentation software on measurement accuracy. **Methods:** CBCT images from eight patients with maxillary defects were used to generate 3D-printed models for volumetric assessment. Two CBCT systems (Largev Smart and Planmeca Promax) were evaluated at three different kVp settings. Volume calculations were conducted using ITK-SNAP version 4.2.2 and 3D Doctor version 4.0 software, while micro-CT served as the gold standard (GS) for comparison. Statistical analysis included a three-way ANOVA to assess the effect of CBCT parameters and software on volumetric accuracy. Additionally, post-hoc Tukey HSD analysis was performed to identify specific differences between kVp groups, and Pearson correlation analysis was used to evaluate consistency with the GS. Significance level was set at *p* < 0.05. **Results:** Higher kVp settings significantly improved volumetric accuracy, with 100 kVp yielding the smallest deviations (−3.77%) from the GS. Tukey HSD analysis revealed significant differences between 60–80 kVp (*p* = 0.008), 60–100 kVp (*p* < 0.001), and 80–100 kVp (*p* = 0.041), confirming the influence of kVp on accuracy. No significant differences were observed between CBCT devices or software programs (*p* > 0.05). A strong positive correlation (r = 0.96) between CBCT-derived and micro-CT volumes confirmed CBCT’s reliability for volumetric assessments (*p* < 0.001). **Conclusions:** CBCT provides accurate volumetric measurements of maxillary defects, particularly at higher kVp settings. These findings support its clinical application for preoperative planning and postoperative evaluation, offering a cost-effective alternative to micro-CT.

## 1. Introduction

Maxillary defects are conditions that affect the upper jaw and can arise from various causes, such as trauma due to accidents or injuries, pathological defects caused by diseases like cancer or infections, congenital anomalies such as cleft lip and palate, or post-surgical defects following the removal of tumors. These defects significantly impact a patient’s aesthetics, oral function, and overall quality of life. Consequently, treatment approaches aim to restore function, aesthetics, and health. The selection of the optimal treatment depends on factors such as the defect’s size, location, and the patient’s overall health and treatment objectives [1]. A multidisciplinary collaboration involving oral surgeons, prosthodontists, speech therapists, and orthodontists is often necessary to achieve successful outcomes. For prosthetic rehabilitation, dentures are used to replace lost structures and restore function. On the other hand, surgical reconstruction techniques, including bone grafts, flaps, and implants, aim to restore the maxillary structure. An essential factor in the success of surgical interventions is the accurate determination of the ideal bone volume before the operation, as it directly impacts the completeness, ease, success, and duration of the procedure [2,3,4,5,6,7].

Traditional radiographic imaging methods have been widely used for diagnostic evaluations and treatment planning in cases requiring alveolar bone grafting. However, these methods have significant limitations, as they provide only two-dimensional (2D) data of inherently three-dimensional (3D) anatomical structures [4]. Additionally, these methods often suffer from technical issues such as image distortion, magnification errors, superimposition of anatomical structures, projection geometry inaccuracies, and positioning problems [8]. To overcome these limitations, computed tomography (CT) emerged as a more advanced imaging tool. Over the past few decades, CT has proven to be a valuable clinical resource for evaluating anatomical structures and pathological processes in maxillary and mandibular anomalies Medical computed tomography (CT) provides high-resolution, high-contrast, and 3D images without the superposition of anatomical structures, making it particularly useful for assessing graft success in alveolar bone grafting [8,9,10].

In recent years, cone-beam computed tomography (CBCT) has emerged as a popular alternative to multi detector CT in dentistry due to its distinct advantages. It is a cost-effective imaging modality, making it more accessible for dental practices, and it offers reduced radiation exposure, enhancing patient safety. CBCT provides submillimeter accuracy and enables the production of high-resolution hard tissue images that are critical for detailed analysis [10,11]. Furthermore, its compatibility with dentistry-specific software allows seamless integration into dental workflows and facilitates precise treatment planning. Despite these benefits, it is essential to validate CBCT measurements against micro-CT, the gold standard (GS) for imaging. Micro-CT provides submicron-accurate images, but its clinical use is restricted due to ultra-high radiation doses and therefore its application is limited to in vitro studies [12,13,14,15,16].

The segmentation process plays a pivotal role in analyzing maxillary defects, as it involves isolating specific structures of interest from surrounding non-relevant tissues to construct three-dimensional (3D) virtual models [4]. These techniques can be categorized into manual, automatic, and semi-automatic methods. While manual segmentation is widely regarded as the most accurate approach, it is time-consuming and may not be practical for extensive datasets. In contrast, automatic segmentation offers speed and efficiency, but its reliability can vary depending on the complexity of the anatomical structures [4,5]. As a compromise, semi-automatic segmentation has gained popularity as the preferred technique, because it combines the efficiency and repeatability of automatic methods with the accuracy and expertise of the operator [3,4,5].

Accurate quantitative assessment of defect dimensions can be achieved by using cone-beam computed tomography (CBCT) [2,3,5,6,7,12,17,18,19,20]; however, only a limited number of studies [13,15] have compared CBCT-based maxillary defect volumetric measurements with micro-CT as the GS, with reference [13] being a cadaver study. Therefore, the primary aim of this study is to evaluate the accuracy and reliability of maxillary defect volume measurements obtained through CBCT in comparison to micro-CT. Furthermore, the present research aims to assess the effectiveness of different CBCT parameters and software programs in calculating defect volumes, relative to the GS, to identify optimal imaging and analysis protocols.

Null hypothesis tested were;

1. Different CBCT devices would not affect the accuracy of maxillary defect volume measurements.
2. Different kilovolt peak (kVp) settings of CBCT devices would not influence the accuracy of maxillary defect volume measurements.
3. There would be no difference in defect volume measurements segmented using different software programs.

## 2. Materials and Methods

The power calculation was based on an assumed effect size of 0.38, a significance level of α = 0.05, and a standard deviation (SD) of 0.25. The estimated SD was derived from prior studies, which indicated low variability in defect volume measurements obtained via CBCT and micro-CT under similar experimental conditions [21]. Given these parameters, a total sample size of 8 patients, each evaluated under three different kVp settings per device, was determined to be sufficient to achieve a statistical power of 0.80. This study was approved by the ethics committees of Ankara University of Faculty of Dentistry (No:36290600/72/2024) and King Abdullah International Medical Research Center (IRB Approval No: 00000101725).

In our study, previously acquired CBCT images from adult eight patients (≥45 years) with unilateral maxillary defects—who had undergone maxillary resection due to cancer and were referred for postoperative evaluation—were utilized. To ensure model accuracy, only patients without metal artifacts (e.g., implants or restorations) in the defect area were included for the creation of 3D-printed models. The DICOM files of these patients were imported into Mimics software (version 22.0; Mimics Innovation Suite; Materialise Medical, Leuven, Belgium). The maxilla regions in the DICOM files were segmented and converted into 3D virtual models. These 3D virtual models were then exported in Standard Tessellation Language (STL) format. The STL files were imported into the Form 2 3D printer (Formlabs Inc., Somerville, MA, USA), and the 3D-printed models were fabricated by using Gray Resin V4 (Formlabs Inc., Somerville, MA, USA), which is a methacrylate-based photopolymer.

Radiographic images of the 3D printed models of 8 patients with maxillary defects were obtained by using two different cone-beam computed tomography (CBCT) systems under three different kVp settings. The Largev Smart 3D CBCT (Largev, Beijing, China) was set to 60 kVp, 80 kVp, and 100 kVp, with 6 mA, a field of view (FOV) of 15 × 9 cm, a voxel size of 0.25 mm, and a slice thickness of 0.75 mm in the multiplanar plane. Similarly, the Planmeca Promax 3D CBCT (Planmeca, Helsinki, Finland) was set to 60 kVp, 80 kVp, and 96 kVp, with 6.3 mA, a FOV of 13 × 9 cm, a voxel size of 0.25 mm, and a slice thickness of 0.75 mm in the multiplanar plane. This resulted in 24 CBCT volumes (8 models × 3 protocols per system), which were saved in DICOM format.

**Figure 1:**
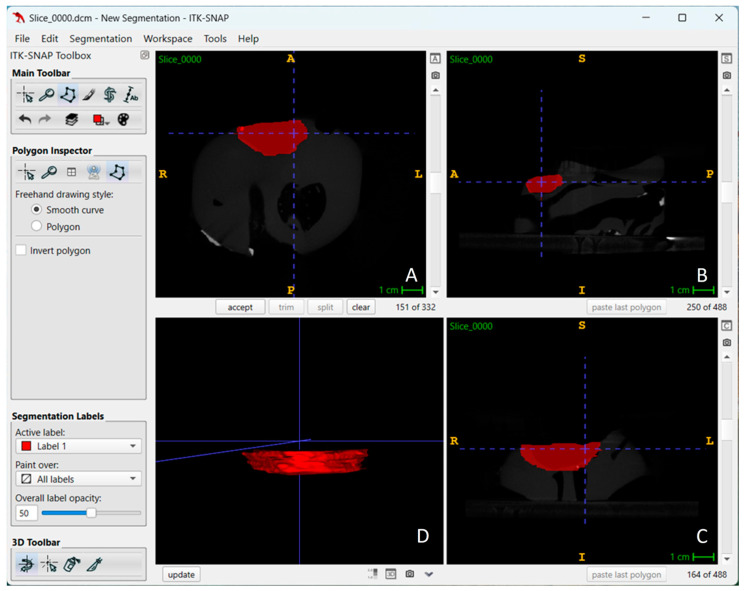
Multi-planar segmentation visualization by using ITK-SNAP version 4.2.2 (**A**) Axial view showing the segmented region of interest (highlighted in red). (**B**) Sagittal view illustrating the placement of the segmented area. (**C**) Coronal view providing a cross-sectional representation of the segmentation. (**D**) 3D reconstruction of the segmented region, highlighting the extracted volume for further analysis.

**Figure 2:**
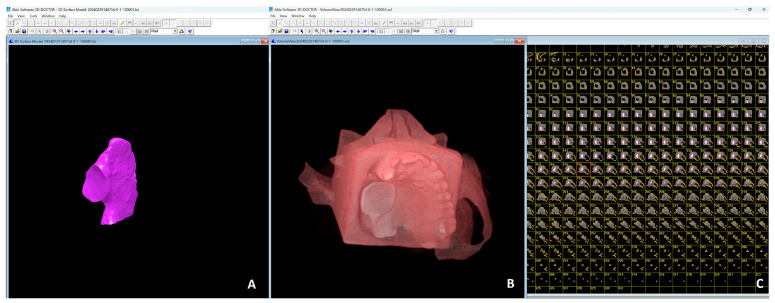
Visualization of segmented 3D models generated using 3D-DOCTOR software. (**A**) Internal volume of the model highlighted in purple. (**B**) 3D reconstructed anatomical model with surface details. (**C**) Corresponding 2D slice images arranged sequentially, used for constructing and analyzing.

**Figure 3:**
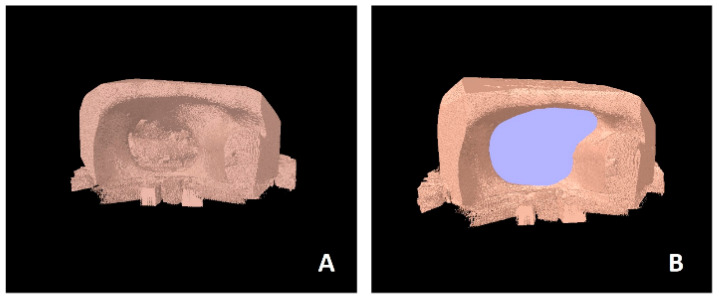
Micro-CT images of defect volume. (**A**) Segmented micro-CT scan data, (**B**) Micro-CT image with digitally highlighted regions in purple to calculate volume of defect. The purple areas represent regions of interest identified during analysis.

All files were then transferred to 3B ITK SNAP (version 4.2.2; www.itksnap.org) (Figure 1) and 3D Doctor Software (version 4.0; Able Software Corp., Lexington, MA, USA) (Figure 2) for volume calculations. These softwares allow defect segmentation on consecutive axial slices, enabling defect visualization at each level apico-coronally. This ensured detailed slice-by-slice segmentation of the defect borders manually using a mouse with color delineation. Automated calculation of the total volume from the areas outlined on each slice of known thickness (0.75 mm) was performed by using software algorithms on the volume measurement screen. A single experienced observer in oral and maxillofacial radiology (KK) performed the measurements twice using a 22″ NEC MD213MG LCD monitor (NEC, Tokyo, Japan) with a resolution of 2048 × 1536 pixels and 32-bit color depth in a darkened room. The averages were recorded.

The models with maxillary defects were also examined using the SkyScan 1272 micro-CT system (Bruker microCT, Kontich, Belgium), which served as the gold standard (GS). Prior to scanning, the 3D-printed models were trimmed to isolate the defect region, allowing them to fit within the sample chamber of the SkyScan 1272. This enabled high-resolution, submicron imaging of the defects for volume validation. Based on pilot studies, the micro-CT technical settings were configured to 90 kVp, 800 mA, a 4-s exposure time, a total scan time of 4 h and 10 min, an angular rotation step of 0.4 degrees, and a voxel size of 26 µm. To standardize the analysis, the region between the two defect margins was measured, and standard regions of interest (ROIs) were used. Histogram equalization was performed using the CTAn software’s (version: 1.13; Bruker microCT, Kontich, Belgium) automatic threshold (Figure 3). Images had a bit depth of 16 bits, and global thresholding was applied for measurements. For accurate segmentation of landmarks, ROI adjustment, and thresholding, a well-trained, calibrated researcher conducted the measurements using the CTAn software’s automatic calibration program tailored to sample thickness. All volume measurements were repeated twice and averaged for precision. All volume measurements were performed twice by a single experienced observer (KK) with a one-week interval. Intra-observer reliability was quantified using an intra-class correlation coefficient (ICC) under an absolute-agreement model, yielding an ICC of 0.98 (95% CI: 0.96–0.99).

### Statistical Analysis

Statistical analyses were conducted using Minitab (version 21; İnova Yazılım, Çankaya, Ankara/SPAC Danışmanlık). A three-way analysis of variance (ANOVA) was performed to assess the effects of the main factors—CBCT device, software, and kVp levels—and their interactions on CBCT-derived volume measurements of maxillary defects, as well as on deviations from the GS volume measurements. Post-hoc comparisons were carried out using the Tukey HSD test to identify specific group differences. Additionally, Pearson correlation coefficients (r) were calculated to assess the relationship between CBCT-derived volume and GS volume measurements, as well as between CBCT-derived volume and differences from GS volume. Statistical significance was set at *p* < 0.05.

## 3. Results

The average volume measurements made on the images obtained from all maxillary defect models and their differences compared to the GS are presented in Table 1, considering 2 different software, 2 different devices, 3 different kVp variables. To evaluate the accuracy of the measured volumes against the GS, percentage deviations were calculated for each group.

A three-way ANOVA was conducted to examine the effects of CBCT device, software, and kVp levels on defect volume measurements (Table 2). No significant main effects were found for CBCT device (*p* = 0.064), software (*p* = 0.079), while significance was found for kVp variables (*p* < 0.001). Post-hoc analyses revealed that significant differences were observed between kVp 60–80 (*p* = 0.008), kVp 60–100 (*p* < 0.001), and kVp 80–100 (*p* = 0.041). Interaction effects among CBCT, software, and kVp were not significant (*p* > 0.05).

A three-way ANOVA was conducted to evaluate the effects of CBCT device, software and kVp levels on volume deviations from GS (Table 3). The results showed that only the kVp levels had a significant effect on volume deviations. (*p* < 0.001). Neither the CBCT device (*p* = 0.435) nor the software (*p* = 0.414) exhibited significant effects. Interaction effects among CBCT device, software, and kVp levels were not significant (*p* > 0.05). Post-hoc analysis revealed that significant differences were observed between kVp 60–80 (*p* = 0.006) and kVp 60–100 (*p* = 0.004). No significant difference was found between kVp 80 and kVp 100 (*p* = 0.234).

To investigate the relationship between the CBCT derived-volumes and their deviations from the GS, Pearson correlation analysis was conducted. The correlation analysis revealed a strong and statistically significant negative correlation between measured volume and deviation values (*r* = −0.88, *p* < 0.001). Moreover, the strong positive linear relationship between the CBCT derived volume and the GS volume, with a Pearson correlation coefficient of 0.96 (*p* < 0.001). This finding highlights the accuracy and reliability of the software in estimating volumes consistent with the GS.

**Figure 4:**
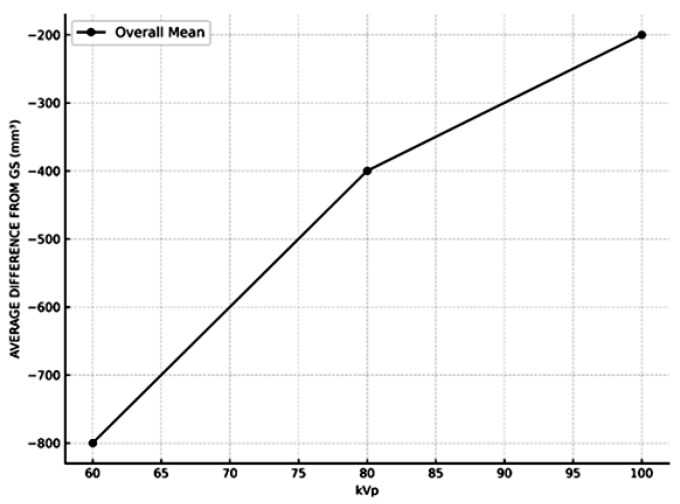
Mean change in volume (mm^3^) with different kVp levels. The graph illustrates the relationship between kVp and the average deviation from the GS.

To evaluate the accuracy of the measured volumes against the GS, percentage deviations were also calculated for each group. It was determined that the measurements closest to the GS in terms of volume values were calculated from the images obtained using the highest kVp of the devices (Figure 4).

## 4. Discussion

We aimed to evaluate the accuracy and reliability of maxillary defect volume measurements obtained using CBCT, in comparison to gold standard (GS) micro-CT measurements. Additionally, we assessed the impact of various factors, including CBCT devices, software programs, and kVp levels, on measurement accuracy.

The accuracy of the segmentation process for evaluating maxillofacial structures depends on several factors, including the CBCT system and its exposure settings, the surrounding structures at the boundaries of the analyzed element, the segmentation software utilized, and the operator’s performance [4,15]. Additionally, the image quality of CBCT scans is affected by factors like the scanning unit and acquisition parameters, including field of view (FOV), tube voltage, tube amperage, and voxel size [10,11]. Although these parameters are critical, there is a limited number of studies in the literature discussing the influence of acquisition protocols on defect volume assessments. Some researchers [2,22,23,24] suggest that voxel size does not affect the accuracy of linear measurements, such as diameter and height. However, for volumetric measurements, smaller voxel sizes enhance the accuracy of defect volume evaluations [25,26,27]. Tolentino et al. [23] conducted linear measurements on CBCT scans with three different voxel sizes by using three different software programs. Their findings revealed that the software programs were reliable and accurate when compared to gold-standard physical measurements, with voxel size showing no significant effect on the accuracy of linear measurements. Similarly, Barbosa et al. [4] reported that acquisition parameters such as FOV and voxel size did not influence the accuracy of defect volume measurements, endorsing the use of low-dose protocols to reduce radiation exposure. In contrast, Dong et al. [25] identified significant variations in volumetric measurements among different voxel sizes. Likewise, Silveira et al. [26] demonstrated that CBCT protocols with varying voxel sizes significantly impacted volumetric measurements in simulated internal root resorptions. They further noted that voxel size might influence accuracy, particularly when assessing small defects [26,27]. In this study, volume measurements were conducted using images acquired with an optimal voxel size of 0.2 mm. Although the maxillary defect volumes evaluated in this research were larger than those typically observed in periodontal defects, intra-bony defects, or root resorption defects, utilizing a consistent optimal voxel size allowed us to emphasize the impact of other factors influencing measurement accuracy. Furthermore, our findings revealed that as defect volume increased, the measurements became more closely aligned with the GS.

The reported accuracy of craniofacial structure segmentation varies, with small field of view (FOV) CBCT devices generally providing greater image contrast compared to larger FOV systems [28]. Larger FOV units often exhibit higher radiation doses, lower contrast and a reduced signal-to-noise ratio, making image segmentation more challenging [16]. Lower-quality images with blurred edges compromise accuracy by obscuring the sharp and clear separation of structures, which is critical for volumetric measurements. Conversely, high-resolution images with sharper edges enhance segmentation accuracy, while low-resolution images with indistinct boundaries impede precise threshold selection [19]. Despite using a relatively large FOV (15 × 9 cm for Largev and 13 × 9 cm for Planmeca Promax) in our study, we observed strong correlations with a Pearson correlation coefficient of 0.96 between the volumetric defect measurements and the GS volumes (*p* < 0.001). This demonstrates that CBCT-derived volumetric measurements are highly consistent with the GS volumes, underscoring their accuracy and reliability. Minimum available FOV offered by the CBCT unit which is able to capture desired defect area is suggested in terms of radiation protection.

Studies investigating the effect of slice thickness on volume estimations of organs or cavities using CT images have reported that increased slice thickness leads to volume underestimations [29,30]. Sezgin et al. [30]. demonstrated that volume estimates using slice thicknesses of 0.2 mm, 0.6 mm, and 1 mm did not significantly deviate from the GS. However, slice thicknesses of 1.4 mm and 2.2 mm significantly underestimated actual volumes due to a loss of sectional detail. In the present study, a slice thickness of 0.75 mm was employed, as it represents the minimum thickness required by the software to merge sections and calculate volume with minimal standard deviation.

It is widely recognized that higher radiographic settings, such as increased milliamperage (mA) and kilovolt peak (kVp), result in higher-quality volumetric images [8,9,10]. In our study, the results demonstrated that kVp significantly influenced the accuracy of CBCT-derived volume measurements (*p* < 0.001). Specifically, higher kVp levels resulted in smaller deviations from the GS, indicating improved measurement accuracy. The measured volumes in our study ranged from 3.77 to 8.47 mm^3^, suggesting that variations in kVp levels contribute to the differences observed in volume measurements. Conversely, the choice of CBCT device and software appeared to have a minimal effect on volumetric measurements. This underscores the need to standardize kVp settings to minimize variability in CBCT-derived volume measurements.

It is important to note that the radiation exposure is inversely related to kVp settings. Higher kVp values, such as 100 kVp, typically lead to lower radiation doses for the same image quality, as higher energy X-rays penetrate tissues more efficiently. However, this must be balanced with image quality requirements. Lower kVp settings (60–80 kVp) may result in higher radiation exposure, but this is often mitigated by adjusting other parameters like mA. In clinical practice, optimizing kVp to achieve high-quality images with minimal radiation is essential, particularly in the context of dental imaging where patient safety is a priority. While we didn’t measure radiation exposure directly, future studies could investigate the effective dose across different kVp settings to provide more precise comparisons.

Micro-CT was used as the gold standard due to its submicron resolution and ability to provide highly accurate volumetric measurements, which is crucial for validating CBCT-derived volumes in this in vitro study. While its field of view is smaller than CBCT, its high resolution and precision ensure reliable and detailed volume assessments. The volumetric assessment of alveolar clefts can be performed using the water displacement technique with 3D-printed models constructed from CT or CBCT images [20,31,32] in a clinical setting. In recent in-vitro studies, the accuracy of volume measurements has been evaluated by comparing CBCT-derived volumes with those obtained from micro-CT [14,15,16]. Ahlowalia et al. [33] assessed the effectiveness of micro-CT and CBCT in measuring bone defect volumes, finding that both methods demonstrated high agreement with measurements obtained using the Archimedes principle. Karavasen et al. [15] reported that CBCT scans underestimated volumes by 4.11% compared to micro-CT values, although this difference was not statistically significant. Similarly, Yavuz et al. [14]. reported that CBCT underestimated the volume of mandibular symphysis bone grafts by 4.53%. Likewise, Liu et al. [16]. observed a similar underestimation of 4.11%, which aligns with the findings of Karavasen et al. [15]. Albuquerque et al. [34] compared the accuracy of multi-slice computed tomography (MSCT) and CBCT in measuring bone defects in cleft palate cases, using a wax model’s real volume calculated via Archimedes’ water displacement technique as the GS. Their results indicated that both MSCT and CBCT provided highly accurate volumetric measurements, with CBCT underestimating defect volumes by 2.4% and MSCT by 1.4%. These differences were not statistically significant, demonstrating that both techniques are reliable for preoperative planning and postoperative assessment of alveolar bone grafts in cleft patients. In contrast, Amirlak et al. [2] evaluated CBCT’s accuracy for volumetric analysis of alveolar cleft defects and simulated bone grafts using water displacement as the GS. Their findings revealed that CBCT slightly overestimated defect volumes, with a mean defect volume of 0.399 mL compared to the actual mean of 0.392 mL, corresponding to a percentage difference of 2.52%. However, no significant differences were observed between CBCT-derived and actual volumes for both grafts and defects. Consistent with these findings, our study observed a slight underestimation in CBCT-derived measurements compared to micro-CT, with an average percentage variation of 5.66%. These results collectively confirm that CBCT provides reliable volume measurements, with minor deviations compared to micro-CT.

Variations in deviation percentages reported among studies may arise from differences in methodologies and in software used. El-Beblawy et al. [18] evaluated the accuracy of formula-based volume calculations (e.g., ellipsoid formula) compared to 3D image segmentation methods using three software programs: MIMICS, OnDemand, and InVesalius. All three programs underestimated defect volumes relative to the GS, with no significant differences among them. However, formula-based calculations significantly underestimated volumes more than software-based methods. Similarly, Kauke et al. [35] reported that the ellipsoid formula underestimated volumes by an average of 10.1% compared to segmentation-based calculations using ITK-SNAP software. On the other hand, Dejaco et al. [36] found that the ellipsoid formula, which calculates volume based on the largest lesion diameter in all three planes, provided a reasonable approximation of head and neck tumor volumes compared to manual slice-by-slice segmentation. Nevertheless, manual and semi-automatic segmentation methods demonstrated superior accuracy and reliability for precise volume assessments. Weissheimer et al. [37] compared six imaging software programs—Mimics, Dolphin3D, ITK-SNAP, OsiriX, InVivo Dental, and OnDemand3D—for upper airway volume measurements. Although all programs were reliable, they showed varying degrees of error in oropharynx volume segmentation. Mimics, Dolphin3D, ITK-SNAP, and OsiriX demonstrated high accuracy with less than 2% error, whereas InVivo Dental and OnDemand3D had errors exceeding 5%. In other studies, Fyllingen et al. [38] reported a 5.5% error for ITK-SNAP in brain tumor volume assessments, while Lee et al. [39] found a precision value of 0.934 ± 0.037 for cerebellar volumetric measurements using ITK-SNAP in magnetic resonance imaging. While Fyllingen et al. [38] and Lee et al. [39] report ITK-SNAP performance in MRI-based neuroanatomical segmentation, they underscore the software’s general reliability and accuracy in complex 3D delineations, reinforcing its suitability for volumetric analysis of maxillary defects in CBCT datasets. In the present study, defect volumes were calculated using manual segmentation with two different software programs. No statistically significant differences were observed between the software programs (*p* > 0.05), highlighting the reliability of both methods for volumetric measurements.

According to the findings of our study, the first null hypothesis—that different CBCT devices will not affect the accuracy of maxillary defect volume measurements—was accepted, as no statistically significant differences were found in defect volumes segmented from different CBCT devices. The second null hypothesis was rejected; as statistically significant differences were observed in defect volume measurements obtained from CBCT images at different kVp settings. Finally, the third null hypothesis was accepted, as no statistically significant differences were observed between analyses conducted using two different software programs.

A limitation of our study is that the use of 3D-printed models based on virtual segmentations does not fully replicate clinical conditions. Factors such as patient movement, variability in head positioning, and the influence of soft tissues were not accounted for in the present study. Additionally, only manual segmentation methods were employed, excluding the potential contributions and efficiency of automated segmentation approaches. Despite these limitations, our study demonstrates the reliability of CBCT for volumetric analysis of maxillary defects, provided that appropriate imaging protocols are meticulously followed. These findings emphasize the importance of standardization in acquisition parameters, particularly in optimizing kVp settings, to enhance measurement accuracy further. Future research should explore the effects of automated segmentation, soft tissue simulation, and real clinical settings to better understand and optimize CBCT accuracy for defect volume assessment.

Accurate volumetric measurement of maxillary defects using CBCT holds significant clinical relevance, particularly in the fabrication of maxillary obturator prostheses. Traditional techniques, such as manual impression-taking, are prone to inaccuracies due to the complex anatomy of maxillary defects. In contrast, a digital workflow enhances both precision and reproducibility compared to conventional methods. Our study demonstrates that CBCT-derived volumetric data are reliable for preoperative planning and prosthetic design, providing a non-invasive, cost-effective, and highly accurate alternative—especially valuable for head and neck cancer patients undergoing extensive resective surgeries [40].

Additionally, optical scanners have gained increasing attention for their ability to capture high-resolution surface detail. However, their performance may be limited in challenging anatomical regions such as deep maxillary defects, where CBCT offers superior penetration and volumetric assessment [41]. In this regard, the three-dimensional data provided by CBCT can complement the surface geometry captured by optical scanners, enabling a more comprehensive approach to prosthetic rehabilitation. This combination has the potential to optimize the digital workflow in maxillary obturator fabrication, enhancing both fit and patient comfort.

## 5. Conclusions

This study evaluated the accuracy and reliability of CBCT-derived maxillary defect volume measurements in comparison to GS micro-CT measurements. Additionally, the effects of CBCT devices, software programs, and kVp settings on volume measurement accuracy were assessed.

Our findings revealed that:

(1) Different CBCT devices did not significantly affect the accuracy of volume measurements, confirming the consistency and reliability of these devices under standard conditions.
(2) kVp settings had a significant effect on measurement accuracy, with higher kVp levels yielding results closer to the GS.
(3) There were no significant differences between volume measurements obtained using the two software programs utilized suggesting their reliability in defect volume estimation.
(4) As defect volume increased, the measurements became more closely aligned with the GS.

## Figures and Tables

**Table 1:** The results of volume measurements and their differences for various combinations of CBCT systems, segmentation software, and kVp settings.

CBCT	Software	kVp	Average Volume Measurement		Average Difference from Gs		Average Percentage Difference from Gs	
			Mean (mm^3^)	Standard Deviation	Mean (mm^3^)	Standard Deviation	Mean (%)	Standard Deviation
**LARGEV**	ITK SNAP	1	9450.88	2250.12	−901.25	200.67	−8.47	3.25
**LARGEV**	ITK SNAP	2	10,673.88	2200.89	−368	190.12	−4.83	2.78
**LARGEV**	ITK SNAP	3	10,198.25	2230.65	−153.88	170.54	−3.85	2.56
**LARGEV**	3D DOC	1	9452	2245.76	−900.12	210.32	−8.38	3.20
**LARGEV**	3D DOC	2	10,673.88	2220.42	−370.25	195.43	−4.72	2.65
**LARGEV**	3D DOC	3	10,200.88	2232.10	−151.25	165.89	−3.77	2.60
**PLANMECA**	ITK SNAP	1	9451.63	2245.12	−900.50	195.87	−8.45	3.18
**PLANMECA**	ITK SNAP	2	10,673.63	2210.32	−370.25	185.42	−4.75	2.70
**PLANMECA**	ITK SNAP	3	10,198	2222.12	−154.13	175.34	−3.80	2.55
**PLANMECA**	3D DOC	1	9453.50	2230.25	−898.62	200.34	−8.42	3.22
**PLANMECA**	3D DOC	2	10,674.63	2240.11	−371.50	190.75	−4.70	2.62
**PLANMECA**	3D DOC	3	10,201.38	2235.32	−150.75	180.21	−3.78	2.58

**Table 2:** Results of the three-way ANOVA for the measured volume values with the factors CBCT device, software, and kVp levels.

Source	Sum of Squares (SS)	df	Mean Square (MS)	F	*p*-Value
**CBCT**	135,820.45	1	135,820.45	4.21	0.064
**SOFTWARE**	142,512.39	1	142,512.39	4.41	0.079
**kVp**	512,230.88	2	256,115.44	7.92	<0.001
**CBCT × SOFTWARE**	32,145.78	1	32,145.78	1.00	0.321
**CBCT × kVp**	42,320.67	2	21,160.33	0.65	0.523
**SOFTWARE × kVp**	45,612.23	2	22,806.12	0.71	0.491
**CBCT × SOFTWARE × kVp**	28,750.12	2	14,375.06	0.45	0.642
**Error**	2,120,034.56	63	33,650.23		
**Total**	2,928,726.28	71			

**Table 3:** Results of the three-way ANOVA for the volume differences from the GS with factors CBCT device, software, and kVp levels.

Source	Sum of Squares (SS)	df	Mean Square (MS)	F	*p*-Value
**CBCT**	124,580.25	1	124,580.25	0.62	0.435
**SOFTWARE**	135,420.12	1	135,420.12	0.67	0.414
**kVp**	215,420.43	2	107,710.22	10.45	<0.001
**CBCT × SOFTWARE**	38,210.45	1	38,210.45	1.87	0.176
**CBCT × kVp**	54,325.34	2	27,162.67	1.32	0.271
**SOFTWARE × kVp**	45,210.67	2	22,605.33	1.10	0.335
**CBCT × SOFTWARE × kVp**	32,120.56	2	16,060.28	0.78	0.462
**Error**	1,412,034.89	63	22,413.25		
**Total**	1,812,345.69	72			

## Data Availability

The datasets used and/or analyzed during the current study are available from the corresponding author on reasonable request.

## References

[1] Beumer J., Curtis T.A., Firtell D.N. (1979). Maxillofacial Rehabilitation: Prosthodontic and Surgical Considerations.

[2] Amirlak B., Tang C.J., Becker D., Palomo J.M., Gosain A.K. (2013). Volumetric analysis of simulated alveolar cleft defects and bone grafts using cone beam computed tomography. Plast. Reconstr. Surg..

[3] Linderup B.W., Küseler A., Jensen J., Cattaneo P.M. (2015). A novel semiautomatic technique for volumetric assessment of the alveolar bone defect using cone beam computed tomography. Cleft Palate Craniofac. J..

[4] de Rezende Barbosa G.L., Wood J.S., Pimenta L.A., Almeida S.M.D., Tyndall D.A. (2016). Comparison of different methods to assess alveolar cleft defects in cone beam CT images. Dentomaxillofac. Radiol..

[5] Janssen N.G., Schreurs R., Bittermann G.K.P., Borstlap W.A., Koole R., Meijer G.J., Maal T.J.J. (2017). A novel semi-automatic segmentation protocol for volumetric assessment of alveolar cleft grafting procedures. J. Craniomaxillofac. Surg..

[6] Jotikasthira P., Chaiworawitkul M., Khwanngern K., Powcharoen W. (2025). Correlation Between Presurgical Alveolar Cleft Volume Measured by Simulation Software Using CBCT and Actual Bone Volume Used for Grafting. Cleft. Palate. Craniofac J..

[7] Stoop C.C., Janssen N.G., Ten Harkel T.C., Rosenberg A.J.W.P. (2023). A Novel and Practical Protocol for Three-Dimensional Assessment of Alveolar Cleft Grafting Procedures. Cleft Palate Craniofac J..

[8] Scarfe W.C., Farman A.G., Sukovic P. (2006). Clinical applications of cone beam computed tomography in dental practice. J. Can. Dent. Assoc..

[9] Angelopoulos C., Scarfe W.C., Farman A.G. (2012). A comparison of maxillofacial CBCT and medical CT. Atlas Oral Maxillofac. Surg. Clin. N. Am..

[10] Kamburoğlu K., Murat S., Kolsuz E., Kurt H., Yüksel S., Paksoy C. (2011). Comparative assessment of subjective image quality of cross-sectional cone-beam computed tomography scans. J. Oral. Sci..

[11] Spin-Neto R., Gotfredsen E., Wenzel A. (2013). Impact of voxel size variation on CBCT-based diagnostic outcome in dentistry: A systematic review. J. Digit. Imaging.

[12] Koç A., Kaya S. (2021). Is it possible to estimate volume of bone defects formed on dry sheep mandibles more practically by secondarily reconstructing section thickness of cone beam computed tomography images?. Dentomaxillofac. Radiol..

[13] Hoang T.H., Nguyen K.T., Kaipatur N.R., Alexiou M., La T.G., Lagravère Vich M.O., Major P.W., Punithakumar K., Lou E.H., Le L.H. (2024). Ultrasonic mapping of midpalatal suture—An ex-vivo study. J. Dent..

[14] Yavuz M.S., Buyukkurt M.C., Tozoglu S., Dagsuyu I.M., Kantarci M. (2009). Evaluation of volumetry and density of mandibular symphysis bone grafts by three-dimensional computed tomography. Dent. Traumatol..

[15] Kasaven C.P., McIntyre G.T., Mossey P.A. (2017). Accuracy of both virtual and printed 3-dimensional models for volumetric measurement of alveolar clefts before grafting with alveolar bone compared with a validated algorithm: A preliminary investigation. Br. J. Oral. Maxillofac. Surg..

[16] Liu Y., Olszewski R., Alexandroni E.S., Enciso R., Xu T., Mah J.K. (2010). The validity of in vivo tooth volume determinations from cone beam computed tomography. Angle Orthod..

[17] Etemadi Sh M., Movahedian Attar B., Mehdizadeh M., Tajmiri G. (2021). Evaluation of the CBCT imaging accuracy in the volumetric assessment of unilateral alveolar cleft. J. Stomatol. Oral. Maxillofac. Surg..

[18] El-Beblawy Y.M., Bakry A.M., Mohamed M.E.A. (2024). Accuracy of formula-based volume and image segmentation-based volume in calculation of preoperative cystic jaw lesions’ volume. Oral. Radiol..

[19] Feng B., Jiang M., Xu X., Li J. (2017). A new method of volumetric assessment of alveolar bone grafting for cleft patients using cone beam computed tomography. Oral. Surg. Oral. Med. Oral. Pathol. Oral. Radiol..

[20] Du F., Li B., Yin N., Cao Y., Wang Y. (2017). Volumetric Analysis of Alveolar Bone Defect Using Three-Dimensional-Printed Models Versus Computer-Aided Engineering. J. Craniofac. Surg..

[21] Ferrare N., Leite A.F., Caracas H.C., de Azevedo R.B., de Melo N.S., de Souza Figueiredo P.T. (2013). Cone-beam computed tomography and microtomography for alveolar bone measurements. Surg. Radiol. Anat..

[22] Costa A.L., Barbosa B.V., Perez-Gomes J.P., Calle A.J., Santamaria M.P., Lopes S.C. (2018). Influence of voxel size on the accuracy of linear measurements of the condyle in images of cone beam computed tomography: A pilot study. J. Clin. Exp. Dent..

[23] Tolentino E.S., Yamashita F.C., de Albuquerque S., Walewski L.A., Iwaki L.C.V., Takeshita W.M., Silva M.C. (2018). Reliability and accuracy of linear measurements in cone-beam computed tomography using different software programs and voxel sizes. J. Conserv. Dent..

[24] Fani S., Moudi E., Haghanifar S., Seyedmajidi S., Poursattar-Bejeh Mir A. (2023). Evaluation of the linear and volumetric measuring changes in different positions in CBCT. Clin. Exp. Dent. Res..

[25] Dong T., Xia L., Cai C., Yuan L., Ye N., Fang B. (2019). Accuracy of in vitro mandibular volumetric measurements from CBCT of different voxel sizes with different segmentation threshold settings. BMC Oral. Health.

[26] da Silveira P.F., Vizzotto M.B., Liedke G.S., da Silveira H.L., Montagner F., da Silveira H.E. (2013). Detection of vertical root fractures by conventional radiographic examination and cone beam computed tomography—an in vitro analysis. Dent. Traumatol..

[27] Pinsky H.M., Dyda S., Pinsky R.W., Misch K.A., Sarment D.P. (2006). Accuracy of three-dimensional measurements using cone-beam CT. Dentomaxillofac. Radiol..

[28] Loubele M., Jacobs R., Maes F., Denis K., White S., Coudyzer W., Lambrichts I., van Steenberghe D., Suetens P. (2008). Image quality vs radiation dose of four cone beam computed tomography scanners. Dentomaxillofac. Radiol..

[29] Sahin B., Mazonakis M., Akan H., Kaplan S., Bek Y. (2008). Dependence of computed tomography volume measurements upon section thickness: An application to human dry skulls. Clin. Anat..

[30] Sezgin O.S., Kayıpmaz S., Sahin B. (2013). The effect of slice thickness on the assessment of bone defect volumes by the Cavalieri principle using cone beam computed tomography. J. Digit. Imaging.

[31] Palankar V., Sattur A., Palankar A., Rajeswari S.R., Thakur S., Desai A.K. (2021). Evaluation of Long-term Stability of Secondary Alveolar Bone Grafts in Cleft Palate Patients Using Multislice Computed Tomography and Three-Dimensional Printed Models: A Prospective Study. J. Pharm. Bioallied Sci..

[32] Phienwej K., Chaiworawitkul M., Jotikasthira D., Khwanngern K., Sriwilas P. (2023). Comparison of preoperative measurement methods of alveolar cleft volume using cone beam computed tomography between computer simulation and water displacement methods. Cleft Palate Craniofac. J..

[33] Ahlowalia M.S., Patel S., Anwar H.M., Cama G., Austin R.S., Wilson R., Mannocci F. (2013). Accuracy of CBCT for volumetric measurement of simulated periapical lesions. Int. Endod. J..

[34] Albuquerque M.A., Gaia B.F., Cavalcanti M.G. (2011). Comparison between multislice and cone-beam computerized tomography in the volumetric assessment of cleft palate. Oral. Surg. Oral. Med. Oral. Pathol. Oral. Radiol. Endod..

[35] Kauke M., Safi A.F., Grandoch A., Nickenig H.J., Zoller J., Kreppel M. (2019). Image segmentation-based volume approximation-volume as a factor in the clinical management of osteolytic jaw lesions. Dentomaxillofac. Radiol..

[36] Dejaco D., Url C., Schartinger V.H., Haug A.K., Fischer N., Riedl D., Posch A., Riechelmann H., Widmann G. (2015). Approximation of head and neck cancer volumes in contrast enhanced CT. Cancer Imaging.

[37] Weissheimer A., Menezes L.M., Sameshima G.T., Enciso R., Pham J., Grauer D. (2012). Imaging software accuracy for 3-dimensional analysis of the upper airway. Am. J. Orthod. Dentofac. Orthop..

[38] Fyllingen E.H., Stensjøen A.L., Berntsen E.M., Solheim O., Reinertsen I. (2016). Glioblastoma Segmentation: Comparison of Three Different Software Packages. PLoS ONE.

[39] Lee D.K., Yoon U., Kwak K., Lee J.M. (2015). Automated Segmentation of Cerebellum Using Brain Mask and Partial Volume Estimation Map. Comput. Math. Methods Med..

[40] Rocchetti V., Cavarra F., Agnone A.M., Loro L., Manna F., Boffano P. (2023). Digital workflow for the intraoral removable prosthesis of head and neck cancer patients. Dent. Cadmos.

[41] Brucoli M., Boffano P., Pezzana A., Corio C., Benech A. (2020). The use of optical scanner for the fabrication of maxillary obturator prostheses. Oral. Maxillofac. Surg..

